# Classifying grains using behaviour-informed machine learning

**DOI:** 10.1038/s41598-022-18250-4

**Published:** 2022-08-17

**Authors:** Sudip Laudari, Benjy Marks, Pierre Rognon

**Affiliations:** Particles and Grains Laboratory, School of Civil Engineering, The University of Sydney, Sydney, NSW 2006 Australia

**Keywords:** Engineering, Physics

## Abstract

Sorting granular materials such as ores, coffee beans, cereals, gravels and pills is essential for applications in mineral processing, agriculture and waste recycling. Existing sorting methods are based on the detection of contrast in grain properties including size, colour, density and chemical composition. However, many grain properties cannot be directly detected in-situ, which significantly impairs sorting efficacy. We show here that a simple neural network can infer contrast in a wide range of grain properties by detecting patterns in their observable kinematics. These properties include grain size, density, stiffness, friction, dissipation and adhesion. This method of classification based on behaviour can significantly widen the range of granular materials that can be sorted. It can similarly be applied to enhance the sorting of other particulate materials including cells and droplets in microfluidic devices.

## Introduction

“To separate the wheat from the chaff” is a significant issue for many industries dealing with granular materials. The aim can be to extract high-grade ores from gangue, to remove defective pills, fruits and nuts, or to separate different waste materials for recycling^1–3^. In mining applications, several mechanical separation methods exist including flotation and rotating tumbler. For some materials, however, these methods are ineffective and sensor-based techniques must be employed. They involve presenting individual grains to a sensor for classification. The challenge is then to classify a vast quantity of moving grains into separate categories.

This classification can be based on measurements of the physical properties of the grains such as size, colour, shape, density and chemical composition by using, amongst others, optical or X-ray sensors^4^. However, poor contrast in these measured properties often means that classification is inaccurate or impossible. A similar classification challenge exists with other particulate materials, for instance when detecting rare pathogenic objects suspended in blood flows, or when sorting droplets and cells in microfluidic devices^5,6^.

Often, the grains to be sorted differ by a range of mechanical properties including elastic modulii, contact friction and adhesion. However, these micro-mechanical properties are not practically measurable while grains are moving, and can therefore not be directly used as a basis for sorting. Nonetheless, they may have some observable influence on the motion of the grains. As a result, a contrast in the physical properties of the grains may be indirectly perceived by detecting a contrast in their trajectories. The experiments and analysis presented by Maier et al. have validated this hypothesis^7^. The tests involved wooden spheres and hemispheres, as well as wax balls and cotton balls falling down a moving conveyor belt. By using a multiple object tracking algorithm, the trajectory of each grain could be measured from area-scanned camera images. Subsequently, a random forest machine learning algorithm was found to enable an efficient grain classification based on some selected features of their trajectories, including the maximum and mean velocities at different locations.

The potential for machine learning to address questions related to granular materials has only recently started to be exploited. For instance, machine learning based on image analysis was proposed to relate third body (the interfacial layer of material between contacting particles) morphology to contact rheology^8^, to relate the grain velocity field to the frictional state of a sheared layer undergoing stick-slip^9^, to enable prediction of the discharge time of granular flows in hoppers^10^, and to infer micromechanical parameters from X-ray imaging^11^. Machine learning methods that do not rely on images were found to enable prediction of seismicity of laboratory-scale earthquakes, specifically of their stick-slip stress time series^12^, and to enable optimal bulk-feeder system selection based on grain micro-mechanical properties^13^. More generally, a Support Vector Machine method was shown to enable the prediction of particle rearrangement from the knowledge of their microstructure in a variety of flowing soft materials^14^.

However, research on machine learning applied to sorting granular materials remains embryonic. In particular, the capabilities of a kinematic-based classification are unexplored beyond the first evidence presented in^7^. This work seeks to further demonstrate the potential of using the kinematic signature of moving grains to indirectly detect contrast in their physical properties.

**Figure 1:**
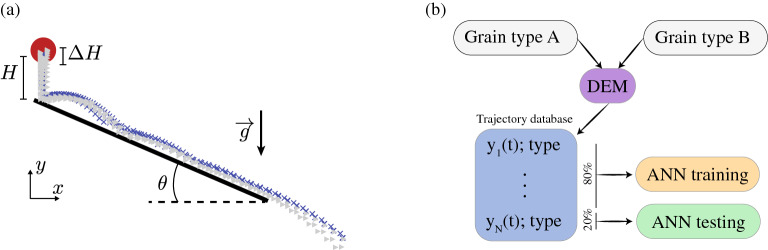
Grain classification based on behaviour. (**a**) Schematic representation of the simulated system where grains are dropped one at the time on an inclined plane. Grains free fall from a height selected at random with the range $H\pm \Delta H$ and bounce or roll onto a planar surface inclined at $\theta =40^\circ$. Symbols represent two families of trajectories corresponding to grains of type *A* ($\times$) and type *B* ($\blacktriangleright$) with different properties. (**b**) Flow chart depicting the method of analysis where an artificial neural network is trained and tested using simulated trajectories.

## Results and discussion

The Discrete Element Method (DEM) code LIGGGHTS^15^ was used to simulate a database of approximately $5\times 10^4$ grain trajectories in the canonical configuration represented in Fig. 1a. Grains free-fall a distance $h\in [H-\Delta H, H+\Delta H]$, and bounce or roll onto an inclined surface. This configuration is chosen for its simplicity and for its potential to yield different trajectories depending on the micro-mechanical properties of the grains.

In the following we seek to achieve a binary classification between grains of type *A* and *B* based on their simulated trajectory. Properties of type *A* grains are summarised on Table 1 and remain constant for all tests. Type *B* grains differ from type *A* by one of these properties, which will be specified below. The initial position *h* of the grains is randomly selected within the range $H\pm \Delta H$, which mimics the effect of a feeder discharging grains into the sensing zone at a random position. As a result, identical grains do not follow a unique trajectory. Rather, each grain type is associated to a family of trajectories. Figure 1a shows that the trajectory families of two grain types may significantly overlap. This means that classifying grains based on simple, explicit criteria such as maximum bounce height and average velocity is not necessarily straightforward or possible.

As an alternative, we have assessed the ability of a machine learning method to achieve this classification. Amongst other possibilities including Random Forests and Support Vector Machine, we chose a simple Artificial Neural Network (ANN); we observed that using a random forest classifier led to similar classification results. The training and testing of the neural network is based on datasets of 100 trajectories of grain type *A* and 100 trajectories of grain type *B*, as illustrated in Fig. 1b. Positions are recorded at a frequency of 100 Hz for a period of two seconds from the release of the particle. As a way to stringently probe the ANN classification ability, the information sent to the network is restricted to the time series of one component of the grain position *y*(*t*). The assumption is that sending additional information such as other position components, velocity and acceleration time series, would only enhance the classification. Using a *k*-fold cross validation, $80\%$ of the trajectories are used for training and $20\%$ for testing. The prediction accuracy of the trained network is measured by the proportion $\epsilon$ of the test trajectories for which the network prediction was correct. The value of $\epsilon$ can take lie between 0 (the network is always wrong) and 1 (the network is always right). A value of $\epsilon =0.5$ is expected for predictions made randomly. It constitutes a benchmark: the network is deemed to have some predicting ability when the accuracy $\epsilon$ is greater than 0.5. Both the DEM and the ANN are well established numerical methods, and their specifics are detailed in “Method” section.

**Figure 2:**
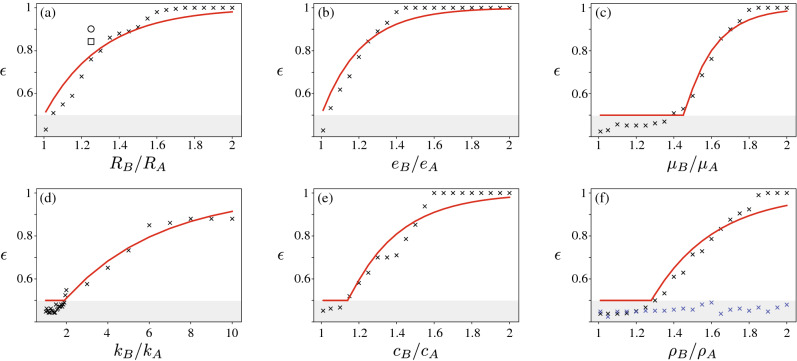
Prediction accuracy $\epsilon$ for grain types with differing properties. (**a**) radius, (**b**) coefficient of restitution, (**c**) coefficient of friction, (**d**) stiffness (**e**) cohesion and (**f**) density without (blue) and with (black) a background drag force (fluid viscosity $\eta =10^{-3}$ Pa s). Each symbol represents the ANN prediction obtained by training and testing against 200 trajectories of two grain types. Properties of grain type *A* are summarised in Table 1. Grains of type B differ by one properties ($\times$), or two properties including coefficient of restitution $e_B =1.25 e_A$ ($\circ$) or coefficient of friction $\mu _B =1.35 \mu _A$ ($\square$). Red lines correspond to best fits of the accuracy using Eqs. (1) or (2). Grey areas denote accuracy lower than that of a random classification ($\epsilon = 0.5$).

Figure 2 depicts the classification accuracy obtained when grains of type *B* differ from grains of type *A* by one of the following properties: radius, coefficient of restitution, coefficient of friction, stiffness, adhesion or density; all other properties being the same. The initial heigh of fall is selected at random in the range 0.12 m $\pm \ 20\%$. The contrast between the two grain types is measured by the ratios $R_B/R_A$, $e_B/e_A$, $\mu _B/\mu _A$ etc. A ratio of 1 means that the two grain types are in fact identical and therefore indistinguishable. A ratio greater than one means that the grain types are different and that there may be different trajectories.

**Table 1:** Properties of type *A* grains: radius *R*, density $\rho$, elastic stiffness *k*, coefficient of restitution *e*, coefficient of friction $\mu$ and cohesion *c*. Type *B* grains feature a difference in one these parameters. Grains are not cohesive ($c=0$) in all tests except for those presented in Fig. 2e.

R [m]	$\rho$ [kg/m$^3$]	k [N/m]	e	$\mu$	c [J/m$^3$]
0.01	2500	$5\times 10^{6}$	0.5	0.4	0 or $10^{4}$

In Fig. 2a the accuracy of the network is shown for grain types featuring a contrast in size (radii $R_A$ and $R_B$). The network accuracy steadily increases from approximately 0.5 in the limit of a size ratio equal to 1, up to nearly 1 when the size ratio is large enough. We propose the following empirical function to capture this trend:

1 $$\begin{aligned} \epsilon (r) = 1- \frac{1}{2} \exp \left( -\frac{r-1}{r_c}\right) , \end{aligned}$$

where *r* is the level of contrast between the two differing properties, in this case $r=R_B/R_A$. The fitting parameter $r_c>1$ characterises the network classification accuracy for a given level of contrast *r*. This exponential function is chosen to match two limits. Firstly, $r=1$ means that grain types are perfectly identical and are therefore indistinguishable: the network accuracy is then expected to be no better than that of a random classification: $\epsilon (r=1) = 0.5$. Secondly, $r\rightarrow \infty$ means that the grain types are so different that the network should be able to correctly classify all grains: $\epsilon (r\rightarrow \infty ) =1$. The choice of an exponential function to interpolate these two limits is arbitrary and other functions could capture the data equally well. The data presented in Fig. 2a are best fit by Eq. (1) using a value of $r_c = 0.33$. Fig. 2b evidences a similar behaviour when grains differ by their coefficient of restitution ($r=e_B/e_A$). The empirical function (1) appears to capture this increase in accuracy with a value of $r_c = 0.16$.

Figure 2c shows a qualitatively different behaviour when the contrasting property is the coefficient of friction. For relatively low ratios $\mu _B/\mu _A$, the accuracy remains at about 0.5, meaning that the network could not detect sufficient differences in the trajectories of the grains to enable classification. The accuracy starts increasing for larger ratios. We propose to capture this by introducing a threshold value $r_t$ greater than 1 into Eq. (1):

2 $$\begin{aligned} \epsilon (r) = {\left\{ \begin{array}{ll} 1- \frac{1}{2} \exp \left( -\frac{r-r_t}{r_c}\right) ,&{} \text {if } r\ge r_t\\ 0.5, &{} \text {otherwise}. \end{array}\right. } \end{aligned}$$

This expression reduces to Eq. (1) for $r_t=1$. The measured accuracy are best fit with $r_t=1.45$ and $r_c = 0.52$. Figure 2d and e display similar behaviour when the contrasting property is the grain stiffness or cohesion, which is best fit by Eq. (2) with $r_t=1.79$, $r_c = 4.93$ and $r_t=1.09$, $r_c = 0.32$ respectively.

In contrast with all the other mechanical parameters, Fig. 2f shows that the accuracy never exceeds 0.5 when grain types have different densities, meaning that classification by density is not possible.

These results indicate that a classification based on grain trajectory is often possible using an ANN. They further show that the prediction accuracy depends on the nature as well as the intensity of the difference in micro-mechanical parameters of the grains. This can qualitatively be explained by the expected influence, or lack of influence, of these parameters on the trajectories. For instance, different grain sizes would impact the effective free fall distance $H-R$ before the first impact. Different coefficients of restitution or different coefficients of friction would lead to different amounts of kinetic energy dissipation at impact. In contrast, grain density does not affect grain free fall in the absence of a background fluid, and does not affect the energy dissipated at impact. With no effect on the grain trajectory, classification is then impossible. To have density influence the grain trajectory, one can introduce a background fluid producing a drag force, such as a Stokes drag $\overrightarrow{F}^{drag} = - 6\pi R \eta \overrightarrow{v}$, where $\eta$ is the fluid viscosity and $\overrightarrow{v}$ the grain velocity. The terminal velocity of a free falling grain is then expected to be given by $\frac{mg}{6\pi R \eta }$, which depends on the density via the grain mass *m*. Figure 2f confirms that density then influences the trajectories in a way that is detectable by the ANN.

**Figure 3:**
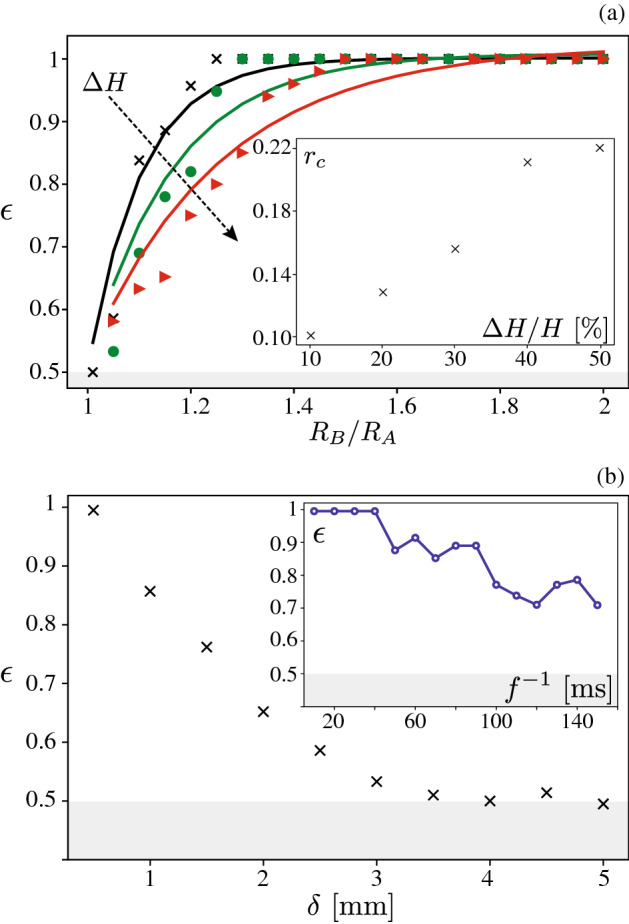
Classification robustness to external noise. (**a**) Classification accuracy obtained for different levels of noise on the initial grain position $\Delta H/H$ = 10% ($\times$), 20% ($\bullet$) and 50% ($\blacktriangleright$) using grain types of differing radius; lines are the best fits using Eq. (1); (inset) corresponding values of the fitting parameter $r_c$. (**b**) Classification accuracy obtained when adding a noise of magnitude $\delta$ on all positions *y*(*t*), or when decreasing the position sampling frequency *f* (inset); results correspond to grain types of differing radii, $R_B=2R_A$ and $\Delta H/H$ = 20%. Grey areas correspond to accuracy lower than 0.5.

The classification accuracy is contingent on the existence of uncontrolled processes influencing the grain trajectory, which we refer to as sources of noise. In the tests presented above, noise was exclusively introduced by randomly selecting the grain initial position within $H \pm \Delta H$. In absence of a noise source, grains from the same type would exhibit identical trajectories. Distinguishing the trajectories of two grain types would then not necessarily require machine learning; it could simply be achieved by using an explicit criterion, for instance by comparing their maximum velocity. A noise source makes classification significantly more challenging, as each grain type is associated to a family of trajectories, as illustrated in Fig. 1a. Whereas a classification based on a single criterion may not be generally possible, machine learning appears to be capable of sensing the effect of micro-mechanical parameters amongst that noise, provided that these effects are sufficiently salient. This qualitatively explains why, for a fixed level of noise $\Delta H$, the classification accuracy increases with the contrast intensity, as observed in Fig. 2. The classification accuracy for a fixed contrast is expected to decrease when the noise is increased. To test this hypothesis, we have repeated the tests presented in Fig. 2a—involving a contrast in radius — for various level of noise $\Delta H$. Figure 3a indicates that the exponential increase in Eq. (1) of the accuracy is preserved at all noise levels; however, the parameter $r_c$ increases approximately linearly with the level of noise $\Delta H$, which reflects how noise hinders classification.

In practice, other factors may impact the classification when measuring the position of the grains using an optical camera and a particle tracking algorithm^7,16^. They include a level of imprecision on the position and a limit to the frequency of image acquisition^17^. To assess the robustness of the classification to these factors, we have mimicked them in our simulated trajectories. Firstly, Fig. 3b shows the classification accuracy obtained when adding random numbers in the range $\pm \delta$ to all positions, considering grain types of differing radii with $r=2$. The classification gradually decreases with an increasing level of noise, but remains close to 1 for sub millimetric noise. Secondly, Fig. 3b shows the effect of the trajectory sampling frequency *f* on the same trajectories using $\delta =0$. The classification accuracy remains close to 1 when the sampling frequency is large enough ($f \gtrsim {25}\hbox { Hz}$, $f^{-1} \lesssim {40}\hbox { ms}$), and decreases at lower frequencies as less and less information are provided to the network. These two results suggest that classification is possible over a range of position sampling rate and precision that are well within the capabilities of standard imaging techniques.

Furthermore, grains may differ by more than one properties. One could expect that having some contrast in more than one property should facilitate classification. To test this hypothesis, we considered grains differing only by their size ($R_B=1.125R_A$) as a benchmark and added a contrast in friction coefficient ($\mu _B=1.135\mu _A$) or in coefficient of restitution ($e_B=1.125e_A$). Results shown of Fig. 2 confirms that additional contrast leads to enhanced classification accuracy.

## Conclusion

“Tell me who you are and I will predict how you behave” is the motto of traditional material modelling. In the context of individual particle sorting we found here that machine learning enables us to reverse this motto to “Tell me how you behave and I will deduce who you are”. This behaviour-based classification can potentially improve a number of sorting processes by indirectly detecting contrast in micro-mechanical properties which would otherwise be difficult to measure. This method should be applicable to processes involving various particulate materials (e.g. grains, droplets, bubbles, cells) moving in various configurations such as down inclined planes, out of silos or through microfluidic devices. The behaviour-based classification, evidenced here for individual grains, could also be applied to classifying bulk materials comprised of many interacting grains, where the dynamics is related to grain micro-mechanical properties^18,19^. Recent advanced in X-ray sensors for measuring the velocity within bulk flows would also enable such classification^17,20,21^.

Remarkably, we found that classification is possible using the simplest network architecture, which involves a single neurone and does not perceive any order in time series. Further still, this network was only provided with minimal information on grain trajectories. It is likely that classification capabilities would be enhanced by using more advanced networks, for instance convolutional networks, and by including more information in their training such as grain velocity and acceleration time series. Furthermore, using machine learning methods with higher interpretability such as random forests could allow for insights on which features of the trajectory are perceived and useful for classification.

**Figure 4:**
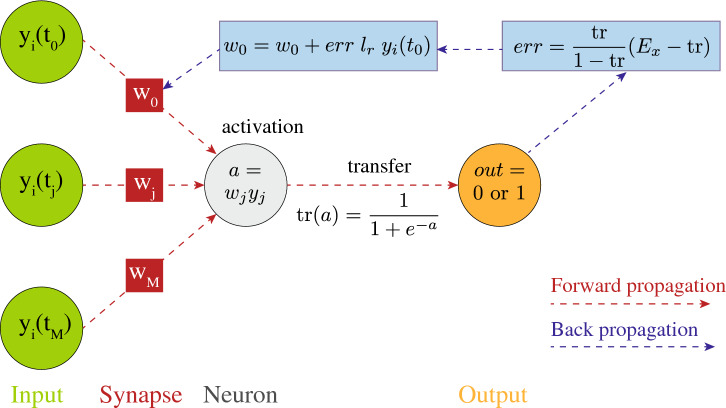
Artificial Neural Network used to classify trajectories, using a feedforward and back propagation training method. The network is comprised of a single neurone and several synapses whose weight *w* evolves during the training to optimise the network prediction. The cycle shown corresponds to one training *epoch*.

## Method

### Discrete element method

DEM consists of a numerical integration of Newton’s second law of motion for individual grain translation and rotation, by discretising them over small time steps *dt*. In our simulations, grains are spherical and are subject to gravity. They interact with the surface by an elasto-dissipative and frictional contact characterised by a normal stiffness *k*, a coefficient of restitution *e* and a coefficient of friction $\mu$. In some instances, an adhesive force following the linear-elastic JKR model is introduced; this adhesive force is proportional the contact surface area *A* via a surface energy term *c*^22,23^. The normal component of the grain-to-surface contact force is $f_n = k\delta _n -\zeta {{\dot{\delta }}}_n -cA$, where $\delta _n$ is the deflection of the contact and $\zeta$ is a viscous parameter yielding some energy dissipation during a normal contact. The tangential contact force involves an elastic component that is capped by a Coulomb friction: $f_t = \min \left( k \delta _t; \;\mu k\delta _n \right)$.

### Artificial neural network

We considered the simple network architecture presented in Fig. 4. It is comprised of a single neurone, which is connected to the input features by synapses. The features are a series of grain positions $y_i(t_j)$. The index *i* refers to a particular grain within a trajectory set; $t_j$ is a particular time at which the grain position was recorded, *M* is the total number of positions presented to the network for one grain trajectory, which is 200 in all the tests but those of Fig. 3b-inset, where the number of features was gradually reduced. The network translates this series of position into a grain type via the weight of each synapse $w_i$, an activation function $a = \sum _i a_iw_i$ and a transfer function $\text {tr}(a) = 1/(1+e^{-a})$ known as the sigmoid function which rescales the activation function between 0 and 1. This result is converted into an output, which is a prediction for the grain type: $\text {tr}\leqslant 0.5$ is interpreted as type A and $\text {tr}>0.5$ type B. The permutative nature of the activation function means that this network is insensitive to the order with which the positions *y*(*t*) are presented.

Training the network consists of identifying a set of weights $w_i$ which leads to some optimal network predictions across a number of trajectories. We use an algorithm known as feedforward-backpropagation to perform the training, which involves the following steps.

Initially, the weights $w_i$ of the network are allocated a random value between 0 and 1. For each trajectory, a feedforward step is applied which consists of using the network to make a prediction on the type of grain. This prediction is compared to the true grain type of the trajectory *j* under consideration, $E_j = 0 \text { or } 1$, via an error function $err= (E_j-\text {tr})*\text {tr}/(1-\text {tr})$. The subsequent back-propagation step uses this error to update the weights according to a stochastic gradient descent method, via the function: $w_i^{new} = w_i^{old} +err\, l_r y_i$, where $l_r$ is referred to as *learning rate*. This feedfowrad-backpropagation cycle is repeated on all trajectories of the training set. This corresponds to one training iteration, commonly referred to as one *epoch*. The two hyper parameters of the training are the number of epochs and the learning rate. We found that using 200 epochs and a learning rate ranging from 0.09 to 0.4 yielded optimum network accuracy while avoiding instances of overfitting. Overfitting is characterised by a training accuracy that steadily improves with the number of epochs while the testing accuracy worsens.
